# Why internet-based education?

**DOI:** 10.3389/fpsyg.2014.01530

**Published:** 2015-01-21

**Authors:** Morton Ann Gernsbacher

**Affiliations:** Department of Psychology, University of Wisconsin-MadisonMadison, WI, USA

**Keywords:** Internet-based learning, online learning, higher education, asynchronous learning, depth of processing, optimal time of day, writing skills

## Abstract

This essay illustrates five ways that Internet-based higher education can capitalize on fundamental principles of learning. Internet-based education can enable better mastery through distributed (shorter, more frequent) practice rather than massed (longer, less frequent) practice; it can optimize performance because it allows students to learn at their peak time of their day; it can deepen memory because it requires cheat-proof assignments and tests; it can promote critical thinking because it necessitates intellectual winnowing and sifting; and it can enhance writing skills by requiring students to write frequently and for a broad audience.

## WHY INTERNET-BASED COURSES?

Over 7 million post-secondary students in the United States – a third of all U.S. college and university students – were enrolled in an Internet-based course last year. Enrollment in Internet-based courses increased a whopping 440% during the past decade (Allen and Seaman, 2014). For the coming decade, most college and university presidents predict that all their students will take an Internet-based course (Parker et al., 2011). Internet-based higher education has moved “from a fad to a fixture” (Selingo, 2013, p. 97).

A decade ago, I volitionally moved all my University of Wisconsin–Madison courses onto the Internet. I wanted to harness the power of the Internet. I also wanted to harness fundamental principles of learning. In this essay, I will illustrate five reasons why Internet-based higher education can capitalize on principles of learning and, therefore, why Internet-based education can be effective pedagogy.

## INTERNET-BASED HIGHER EDUCATION CAN LEAD TO BETTER MASTERY

A core principle of learning is that shorter, more-frequent episodes of practice lead to better mastery than longer, less-frequent episodes (Ruch, 1928; Oseas and Underwood, 1952; Underwood, 1961). Acquiring skills through more frequent practice is considered distributed learning, whereas acquiring skills through less frequent practice is considered massed learning. Distributed learning almost always trumps massed learning (Benjamin and Tullis, 2010).

Distributed learning’s advantage over massed learning has been demonstrated for students of all ages (e.g., Seabrook et al., 2005), acquiring mastery in a wide range of courses (e.g., college composition, Kellogg and Raulerson, 2007; college biology, Reynolds and Glaser, 1964; and college statistics, Budé et al., 2010). Harness the pedagogical power of distributed learning has been one of the most common battle cries for improving higher education (e.g., Willingham, 2002; Roediger and Pyc, 2012).

Internet-based higher education enables more frequent engagement with the material than traditional face-to-face higher education (Holzinger et al., 2009). For example, at my university most face-to-face undergraduate classes meet only twice a week. Many seminar-style courses, including graduate-level courses, meet only once a week. While we professors would like to believe that our students continue to practice their skills when they are not in class, many students wait until the night before class meets to engage with the material. The students then attend class, but several days if not a week pass before the students engage with the material again.

In contrast, Internet-based courses can and should be constructed to require students to engage with the material every day (Newlin and Wang, 2002; Elvers et al., 2003). For example, in my Internet-based courses, students are required to log in almost daily and to complete multiple, small assignments each week (e.g., Foertsch and Gernsbacher, 2008; Gernsbacher, 2013). Furthermore, the assignments are constructed so that it is not in the students’ best interest to mass their practice and attempt to do a week’s worth of assignments in one sitting. Doing so would be akin to trying to do a week’s worth of athletic workouts in one trip to the gym; trying to eat a week’s worth of food in one sitting; trying to visit five European cities in 1 day. It simply would not be feasible.

Thus, Internet-based higher education enables students to distribute their learning over time, to engage with the material in short, frequent episodes, and to master the material in increments, rather than in once- or twice-a-week doses. These short, frequent, and distributed episodes of practice can lead to better mastery.

## INTERNET-BASED HIGHER EDUCATION CAN OPTIMIZE PERFORMANCE

Psychological science, as well as personal observation, identifies differences among us in our optimal time of the day. Our cognitive processes peak at our optimal times and flounder at our non-optimal times (May and Hasher, 1998; May, 1999). Empirical research documents that every cognitive process – memory (West et al., 2002), attention (May, 1999), language comprehension (Natale and Lorenzetti, 1997), even intelligence testing (Goldstein et al., 2007), and attitude change (Martin and Marrington, 2005) – operates at a peak during our optimal time of the day.

The older we get, the earlier in the day we find our peak time for performance (May et al., 1993), which might explain why, at least at my university, many professors like to teach at 8:00 AM. However, at 8:00 AM most traditional-age undergraduate students have barely gone through two full stages of REM sleep (Randall, 2012). Even if students have tried to get a good night of sleep, their biology dictates against morning hours bringing their optimal performance (American Academy of Pediatrics, 2014). Indeed, by puberty, students’ optimal time of the day has already shifted beyond the traditional school day to evening (Kim et al., 2002).

The beauty of Internet-based higher education is that learners can engage with the material – and the course – at whatever time of the day or night works best for them. For example, in my Internet-based courses, all assignments are due at 11:59 PM, but students can complete the assignments hours or days before they are due. Students can also engage with the material around the clock (i.e., 24/7). Thus, Internet-based higher education can optimize performance by allowing students to capitalize on their own optimal time of the day.

## INTERNET-BASED HIGHER EDUCATION CAN DEEPEN MEMORY

Psychological science documents the value of deeper levels of processing (Craik, 2010). Information that is processed to a deeper level is remembered better; more deeply processed information is also more tightly connected to previously learned and subsequently learned concepts. Internet-based learning can deepen levels of processing for one simple reason: To allay concerns about cheating, assignments and exams must assess deeper levels of processing.

One of the primary concerns that faculty have about Internet-based teaching is the worry that students will cheat (Parker et al., 2011). By cheat, instructors usually mean look up the answers. But if the answer to a question, or the solution to a problem, is just a click away – be the assignment Internet-based or in-person – that assignment is not assessing a very deep level of processing. We should probably not assess such superficial knowledge in our higher education courses.

Therefore, in my Internet-based courses, I expect students to take advantage of all the material the world wide web has to offer. I encourage students to click and scroll and open as many browser windows as they want when they are completing assignments, solving problems, and taking exams. If the answer to one of my test questions is just one click away, it is not a very good test question.

Similarly, if instructors hesitant-to-embrace Internet-based instruction are worried about their students enlisting a ringer to complete their assignments or take their tests, my response is the same: do not design assessments that anyone can simply parachute into – regardless of whether you are designing assessments that are Internet-based or in person. Valid assessments should assay mastery of the course material, for which active members of the course should be advantaged.

## INTERNET-BASED HIGHER EDUCATION CAN PROMOTE CRITICAL THINKING

A few years ago, a group of psychology students at the University of Cincinnati refused to spend $168 to purchase the textbook for their course. Instead they gathered all the information they needed for their course using only the Internet. How did these students fare? Top of the class (Massis, 2013).

How could that be? Isn’t the Internet is full of cat videos? Yes, it is (Clark, 2012). But the Internet is also full of 100s of videos that explain how to compute a *t*-test, which is a basic statistical tool for students and scholars. The videos available on YouTube and other Internet-based video sharing sites provide a vibrant component of many curricula, including health education (Akagia, 2008; Burke and Snyder, 2008), African American studies (White, 2009), anatomy (Jaffar, 2012), Shakespeare (Desmet, 2009), music instruction (Kruse and Veblen, 2012), American history (Rees, 2008), and nursing (Clifton and Mann, 2011).

Moreover, as research published in *Nature* demonstrated, information available on the Internet-based Wikipedia is just as accurate as information available in the print-based *Encyclopedia Britannica* (Giles, 2005). That is not to say that either Wikipedia or *Encyclopedia Britannica* is 100% accurate, but Wikipedia is no less accurate than a traditional print-based encyclopedia.

The accuracy of information on the Internet, although commonly underestimated, is one factor that led to the University of Cincinnati students’ success with substituting Internet-based information for a standard textbook. The other factor was that the process of gathering information from the Internet evokes more critical thinking than simply reading a textbook. Active learning – winnowing and sifting intellectual wheat from chaff – facilitates learning (Tsui, 1999; Prince, 2004; Chi, 2009; Freeman et al., 2014). The Internet magnifies the opportunities for winnowing and sifting (Newlin and Wang, 2002; Weiler, 2004).

## INTERNET-BASED HIGHER EDUCATION CAN ENHANCE WRITING SKILLS

After critical thinking skills, writing skills are what employees consistently rank as necessary in college graduates [Association of American Colleges and Universities and Hart Research Associates (AAC&U), 2013; Sternberg, 2013]. However, many college-level instructors rate their students’ writing skills as only fair (Purcell et al., 2013). Internet-based higher education can enhance students’ writing skills by capitalizing on the Internet’s inherently text-based mode of communication (Gernsbacher, 2014) and the Internet’s inherently broad-based audience (Ellison and Wu, 2008).

For example, across one term of my Internet-based courses, each student composes approximately 85 posts, with each post comprising two to three paragraphs. In essence, each student writes the equivalent of a five-page double-spaced paper each of 15 weeks. Text-based communication on the Internet is a feature, not a bug (Gernsbacher, 2014).

Who reads the equivalent of 50 students’ five-page papers each week? I read a sample of them, but the primary readers are the other students in the class. Across the semester, each student reads and comments on over 700 posts written by their peers. Requiring this quantity of reading and writing in a face-to-face college course would consume all the class meeting time. That is not a concern with Internet-based courses.

Moreover, as the Stanford Study of Writing attests (ssw.stanford.edu), today’s Internet-native students are vastly more experienced writing for the public than we professors were at their age (Fishman et al., 2005). Many of today’s college students have written blogs since they were 12 years old and posted Facebook statuses since they were 14; they might have commented on more Internet sites than most professors have read (Keller, 2009).

Therefore, today’s students are “almost always less enthusiastic about their in-class writing because it ha(s) no audience but the professor” and it fails to “serve any purpose other than to get them a grade” (Thompson, 2009). Writing to an audience that comprises only the professor is not a concern with Internet-based courses. Posting on a discussion board is de rigueur in most all Internet-based courses, and attaching a document to a common discussion board for all class members to read is just as easy as emailing it to the professor.

Writing to a broad audience (an entire class or an entire Internet) rather than only the professor empirically improves technical aspects of composition (Day et al., 1998); encourages students to write longer and more often (Kaplan et al., 2007); and increases students’ mastery of logical, ethical, and emotional appeal, as well as increasing their treatment of opposing views (Gaddis et al., 2000).

The Stanford Study of Writing also points to the fact that text-speak rarely if ever enters into students’ course-based writing, an observation I, too, have made. In fact, writing for the Internet increases, rather than decreases, students’ grammatical and syntactic skills (Gaddis et al., 2000).

## IN SUM

This essay illustrates five reasons why Internet-based higher education can capitalize on principles of learning and, therefore, why Internet-based education can lead to effective pedagogy. Internet-based education can lead to better mastery by providing short, frequent episodes of practice rather than less frequent bouts of practice. Internet-based education can optimize performance by allowing students to engage with the material – and the course – at whatever time of the day works best for them.

Internet-based education can deepen memory by necessitating cheat-proof assignments and exams that engage deeper levels of processing. Internet-based education can promote critical thinking by empowering students to gather multiple sources of information and distinguish wheat from chaff. Lastly, Internet-based education can enhance writing skills by multiplying the writing opportunities with a built-in audience beyond the professor.

A recent meta-analysis by the U. S. Department of Education (2010) evaluated 50 high quality contrasts of Internet-based versus face-to-face courses. The results showed a consistent advantage in student learning from Internet-based higher education courses. However, the report cautioned that the “positive effects (of Internet-based learning) should not be attributed to the media, *per se*” (p. ix). Indeed, it is likely that any medium will lead to more successful pedagogy if it capitalizes on fundamental principles of learning.

## Conflict of Interest Statement

The author declares that the research was conducted in the absence of any commercial or financial relationships that could be construed as a potential conflict of interest.
